# In Vivo Targeting of ADAM9 Gene Expression Using Lentivirus-Delivered shRNA Suppresses Prostate Cancer Growth by Regulating REG4 Dependent Cell Cycle Progression

**DOI:** 10.1371/journal.pone.0053795

**Published:** 2013-01-16

**Authors:** Che-Ming Liu, Chia-Ling Hsieh, Yun-Chi He, Sen-Jei Lo, Ji-An Liang, Teng-Fu Hsieh, Sajni Josson, Leland W. K. Chung, Mien-Chie Hung, Shian-Ying Sung

**Affiliations:** 1 Ph.D. Program for Cancer Biology and Drug Discovery, China Medical University and Academia Sinica, Taichung, Taiwan, ROC; 2 Graduate Institutes of Cancer Biology, China Medical University, Taichung, Taiwan, ROC; 3 Graduate Institute of Clinical Medical Science, China Medical University, Taichung, Taiwan, ROC; 4 Center for Molecular Medicine, China Medical University Hospital, Taichung, Taiwan, ROC; 5 Division of Radiation Oncology, China Medical University Hospital, Taichung, Taiwan, ROC; 6 Division of Urology, Buddhist Tzu-Chi General Hospital, Taichung Branch, Taichung, Taiwan, ROC; 7 Department of Urology, School of Medicine, Buddhist Tzu Chi University, Hualien, Taiwan, ROC; 8 Department of Medicine, Cedars Sinai Medical Center, Los Angeles, California, United States of America; 9 Department of Biotechnology, Asia University, Wufeng, Taichung, Taiwan, ROC; 10 Department of Molecular and Cellular Oncology, M.D. Anderson Cancer Center, University of Texas, Houston, Texas, United States of America; Florida International University, United States of America

## Abstract

Cancer cells respond to stress by activating a variety of survival signaling pathways. A disintegrin and metalloproteinase (ADAM) 9 is upregulated during cancer progression and hormone therapy, functioning in part through an increase in reactive oxygen species. Here, we present *in vitro* and *in vivo* evidence that therapeutic targeting of ADAM9 gene expression by lentivirus-delivered small hairpin RNA (shRNA) significantly inhibited proliferation of human prostate cancer cell lines and blocked tumor growth in a murine model of prostate cancer bone metastasis. Cell cycle studies confirmed an increase in the G1-phase and decrease in the S-phase population of cancer cells under starvation stress conditions, which correlated with elevated intracellular superoxide levels. Microarray data showed significantly decreased levels of regenerating islet-derived family member 4 (REG4) expression in prostate cancer cells with knockdown of ADAM9 gene expression. This REG4 downregulation also resulted in induction of expression of p21^Cip1/WAF1^, which negatively regulates cyclin D1 and blocks the G1/S transition. Our data reveal a novel molecular mechanism of ADAM9 in the regulation of prostate cancer cell proliferation, and suggests a combined modality of ADAM9 shRNA gene therapy and cytotoxic agents for hormone refractory and bone metastatic prostate cancer.

## Introduction

Occurring in more than 80% of advanced-stage prostate cancer cases, skeletal metastases correlates with a high level of morbidity; a 5 year survival rate of 25% and median survival of approximately 40 months [1]. Skeletal metastases, due to the development of bone pain, cancer-associated bone fractures and spinal compression, as well as cranial neuropathy, anemia and infection, can significantly compromise the quality of life of prostate cancer patients [2], [3]. Currently, androgen deprivation is the first line of therapy for metastatic prostate cancer; however, prostate cancer will often progress to an androgen-independent bone-metastatic stage. Once this progression occurs, chemotherapy and radiotherapy are the main therapeutic options, both of which cause unpleasant side effects and only provide a limited benefit to the quantity and quality of life [4]. Hence, it is important to pursue new therapeutic factors that may have the potential to improve survival of patients with hormone refractory and bone metastatic prostate cancer.

Despite recent advances in therapeutic strategies, many malignant cancers still develop resistance to radiation and targeted therapies [5], [6]. Resistance occurs as a result of the stress response, allowing malignant cells to overcome the cytotoxic effect of many therapies [7]. A disintegrin and metalloproteinase (ADAM) 9 is an important member of a disintegrin and metalloproteinase gene family. The proteins encoded by this family mediate cellular responses to environmental stress by interacting with a variety of cell surface proteins and regulating diverse cellular processes including proliferation, extracellular matrix binding, and ectodomain shedding [8]–[12]. Previous work done by our group [13] and others [14] have shown in clinical studies that higher ADAM9 levels correlate with a shorter period of prostate cancer remission. We also demonstrated a significant correlation between tumor ADAM9 staining and the risk of prostate cancer recurrence and death in patients who underwent hormone therapy, suggesting that a progressive increase in ADAM9 expression could be used as a biomarker for poor prognosis in prostate cancer patients after hormone therapy [15]. Moreover, knockdown of ADAM9 expression results in increased radiosensitivity and chemosensitivity to therapeutic agents [16], indicating that ADAM9 overexpression by cancer cells might be potential escape mechanism for overcoming stress-induced cancer cell death; however, little is known about the downstream regulatory mechanisms by which ADAM9 promotes cancer cell survival in response to stress. Since elevated ADAM9 expression is observed in many advanced tumors, this raises the possibility that ADAM9 might be a potential biomarker for cancer targeted gene therapy, although more research is necessary.

In the present study, we assess the feasibility of lentiviral vector-delivered small hairpin RNA (shRNA) against ADAM9 for the treatment of androgen-independent and bone metastatic human prostate cancer in an experimental animal model. The molecular mechanism underlying the therapeutic action of ADAM9 targeted gene therapy was also elucidated.

## Materials and Methods

### Materials

Retroviral vectors containing shRNA that targets ADAM9 and control shRNA were obtained from Open Biosystems (Lafayette, CO). Lentiviral vector ADAM9 shRNA and controls were obtained from the National RNAi Core Facility at the Institute of Molecular Biology/Genomic Research Center, Academia Sinica, Taiwan. The anti-ADAM9 antibodies were obtained from R&D Systems (Minneapolis, MN). Anti-human EF1-α obtained from Millipore (Billerica, MA) was used as a control antibody.

### Cell Cultures

The androgen-independent metastatic prostate cancer cell line, PC3 and androgen-dependent prostate cancer cell line, LNCaP, were used in previous studies [13], [17] and cultured in T-medium (Invitrogen, Carlsbad, CA) supplemented with 5% heat-inactivated fetal bovine serum (FBS) (Hyclone, Logan, UT), 50 IU/mL penicillin and 50 µg/mL streptomycin (Invitrogen) and maintained in 5% CO_2_ at 37°C.

### Vectors and RNA Interference

The retroviral plasmid pSM2C and shRNA specific for ADAM9, as well as the control plasmid pGIPZ and shRNA specifically targeting REG4 were obtained from Open Biosystems. The lentiviral pLKO, control shGFP, and shRNA targeting the mRNA of the ADAM9 coding sequence were obtained from the National RNAi Core Facility at the Institute of Molecular Biology/Genomic Research Center, Academia Sinica, supported by the National Research Program for Genomic Medicine Grants of NSC (NSC 97-3112-B-001-016). Target information is listed in figure S1. These shRNA vectors were constructed by inserting annealed oligonucleotides containing the shRNA sequence into EcoRI and AgeI sites downstream of the U6 promoter in the pLKO.1 vector. Recombinant lentivirus carrying shRNA was produced by co-transfecting 293FT cells with a mixture of plasmid DNA consisting of pMD-G (VSV-G envelope), pCMV-ψR8.91 (Gag/Pol/Rev), and pLKO/1-shRNA vectors using TurboFect reagent (Fermentas, Glen Burnie, MA) according to the manufacturer’s recommendations. Virus-containing culture supernatants were collected 2 days after transfection and used to infect PC3, LNCaP, and RCC52 cells in combination with 8 µg/mL polybrene (Sigma-Aldrich, St. Louis, MO). Stable cell lines were selected by culturing cells in 2.5 µg/mL puromycin (Calbiochem, La Jolla, CA) for one week. Western blot, FACS, or qPCR was used to determine the effects of gene expression knockdown.

### Protein Detection

Protein extracts from the cell lines were analyzed on SDS-polyacrylamide gels (15 µg per lane) and transferred to Hybone ECL nitrocellulose membranes (GE Healthcare Life Science, Piscataway, NJ). Blots were probed with monoclonal mouse anti-human antibodies against ADAM9 (R&D systems), p21, and p27 (kind gift from Dr. Yun-Lung Yu at China Medical University & Hospital) according to the manufacturer’s instructions. BT474 cell lysate (cells were treated with 500 nM doxorubicin overnight) was obtained from Dr. Wei-Chien Huang at China Medical University & Hospital. For loading control, blots were probed with an anti-EF1-α monoclonal antibody (1∶10,000; Millipore). After incubation with an HRP-conjugated secondary antibody (1∶5000; GE Healthcare Life Science), chemiluminescent signals were detected using an ECL Plus kit and the blots were exposed to Hyperfilm ECL (GE Healthcare Life Science). Protein band quantification was carried out using ImageJ software (http://rsbweb.nih.gov/ij/, NIH, Bethesda, USA).

### Measurement of Reactive Oxygen Species

Cells were seeded on dishes at 70–80% confluence and starved overnight. For quantification analysis, cells were trypsinized, centrifuged, and resuspended in HBSS with or without 5% FBS (depending on whether they were exposed to radiation or starvation). Hydrogen peroxide was detected using dichlorofluorescindiacetate (DCF, 2 µM) (Invitrogen). Superoxide was detected using dihydroethidium (DHE, 10 µM) (Invitrogen). Superoxide imaging was detected using MitoSox Red mitochondrial superoxide indicator (Invitrogen). Samples were incubated for 40 min at room temperature in the dark on a rotator. Analysis of DCF and DHE fluorescence was performed using a FACSCalibur (BD Biosciences, San Jose, CA) according to manufacturer’s instructions. For imaging mitochondrial superoxide generation, cells were starved for 24 hours followed by loading with MitoSOX Red, Alexa-488 WGA and DAPI (Invitrogen) for 30 min and viewing under a fluorescence microscope.

### Cell Proliferation Assay

The effect of ADAM9 shRNA on cell proliferation was measured by directly counting the number of cells. Briefly, cells were plated at a density of 1×10^5^ on 60-mm dish. At designated times, the cells were removed by trypsinization, and the number of viable cells was counted in a hemocytometer with the use of trypan blue (0.4%) staining.

### Clonogenic Assay

For the clonogenic cell proliferation assay, cell suspensions were prepared by treatment with 0.25% trypsin and 0.05% EDTA. After calculating cell concentration using a hemocytometer, 100 cells were seeded in 6-well plates with T-medium and 5% FBS for 2 weeks. The number of colonies in each well was determined by counting the number of cells in each colony. Only colonies with ≥50 cells were defined as successful. To identify colonies, medium was withdrawn and 3.7% formaldehyde was added for 15 minutes, followed by incubation in 1 mL crystal violet for 15 minutes at room temperature. Colony images were taken and cell numbers calculated using ImgaeJ.

### Transwell Invasion Assay

The invasiveness of cancer cells was assessed using 24-well Transwell (Corning, Lowell, MA) plates. In brief, 2×10^5^ cells in media containing 0.5% FBS were added to the upper chamber containing 8 µm pore polycarbonate coated with 1 mg/mL matrigel; the lower chamber was filled with media containing 5% FBS. After 16 h incubation, the upper surface of the membrane was scrubbed with a cotton-tipped swab. The invading cells on the lower surface of the membrane were fixed and stained with 0.5% crystal violet. Random fields (5 per membrane) were photographed at 40x magnification for calculating cell number. In addition, cells were quantified by measuring the absorbance of dye extracts at 570 nm in 100 µl of Sorenson’s solution (9 mg tirsodium citrate, 305 mL distilled water, 195 mL 0.1 N HCl, 500 mL 90% ethanol).

### Wound Healing Assay

Cells resuspended in culture medium were seeded into 24-well plates. A single wound was created in the center of the cell monolayer by gentle removal of the attached cells with a sterile plastic pipet tip after cell cultures reached ≥90% confluence. The debris was removed by washing with serum-free media. Cells that migrated into the wounded area or those protruding from the border of the wound were visualized and photographed under a Zeiss Axioplan microscope (Carl Zeiss MicroImaging, Thornwood, NY) with a 10× objective at five preselected time points (0, 2, 4, 6, and 8 h). Each experiment was independently performed at least three times.

### Immunohistochemical Staining

Five-micron thick paraffin-embedded tissue sections were deparaffinized and rehydrated. The tissue sections were incubated for 2 hours with mouse monoclonal anti-human ADAM9 antibody (1∶50 dilution, R & D Systems). After washing to remove unbound primary antibody, sections were treated with a dextran polymer backbone conjugated to secondary antibodies and labeled with horseradish peroxidase according to manufacturer’s instructions (DAKO Envision system for mouse and rabbit primary antibodies, DAKO Corporation, Carpinteria, CA) for 30 minutes. Tissue sections were incubated in the chromogenic peroxidase substrate, diaminobenzidine, for 5 minutes; sections were then counterstained with automation hematoxylin (DAKO) for 15 minutes. The specificity of labeling by this procedure was verified by negative control reactions using buffer to replace the primary antibody and isotype-specific IgG. Masson’s trichrome staining was performed using a Masson’s trichrome stain kit (Sigma-Aldrich) following the standard protocol provided by the manufacturer.

### Tartrate-resistant Acid Phosphatase (TRAcP) Staining of Bone

Immunostaining with TRAcP was carried out according to the manufacturer’s protocol (Takara Bio Inc, Shiga, Japan). Briefly, paraffin-embedded mouse tibia bone sections were deparaffinized and rehydrated for the TRAc Posteolytic reaction. The bone sections were incubated with the substrate solution (0.1 volume of sodium tartrate in Naphthol-AS-BI-phosphate/Fast Red Violet LB substrate solution, pH 5.2) at 37°C for 45 minutes. After washing the slide with distilled water and adding glycerol to prevent dehydration, the samples were examined under the microscope.

### Determination of the Cellular DNA Content by FACS Analysis

Cancer cells were plated at 1×10^6^ cells per 100 mm dish and starved for either 24 or 48 hours. Cells were tryspinized and subjected to cell cycle analysis using propidium iodide as reported previously [18]. The relative DNA content of the nuclei was measured by FACSCalibur (BD Bioscience).

### *In Vivo* Xenograft Models

Five-week-old male athymic nude (nu/nu) mice obtained from National Laboratory Animal Center at Taiwan were used for subcutaneous (s.c.), intracardiac, and intratibial tumor implantation. Cells were cultured to 100% confluence, trypsinized and enumerated. Mice were sedated with 1.7% isoflurane mixed with air for s.c. tumor implantation. Xenograft tumors were established by s.c. injection of 10^6^ PC3 cells expressing pSM2c or shADAM9 cells in 100 µL of PBS into both sides of each mouse. Animals were sacrificed after 8 weeks. There were no significant body weight differences between the animals with and without s.c. tumors at the time of sacrifice. For intratibial tumor injection, a 27-gauge needle was used to bore a hole in the marrow cavity through the tibial plateau into both the left and right tibias. A 28-gauge Hamilton syringe was used to inject 5×10^5^ cells in a 50 µL volume into the marrow cavity. Tumor growth was monitored by both bioluminescence and x-rays once per week. Animals were sacrificed after 8 weeks. Tibias were removed, washed in PBS and fixed in 10% formaldehyde at room temperature for 24 h. Bone specimens were washed with PBS followed by decalcification with 0.25 M EDTA in PBS (pH 7.4) at room temperature for 6 weeks. The EDTA decalcification solutions were changed weekly. Tumor growth was monitored weekly using a bioluminescence imaging system (Xenogen IVIS 200 Series, Caliper, Hopkinton, MA).

### RNA Extraction and DNA Microarray Analysis

Total RNA was isolated from cultured cells (Roche Applied Science, Mannheim, Germany) following the manufacturer’s protocol. Purified RNA samples were submitted to Phalanx Biotech for microarray analysis using the Human Whole Genome OneArray® Microarray v5 (Phalanx Biotech Group, Inc., Hsinchu, Taiwan). Each microarray contains 29,187 human genome probes and 1088 experimental control probes. Detailed description of microarray procedures and whole genome gene & probe lists are available from http://www.phalanxbiotech.com/tech_support/general.php.

### Overexpression of REG4

The full length REG4 coding region was amplified by PCR using the primers listed in Materials and Methods S1, and subcloned into p3XFLAG-CMV-7.1 (Stratagene, Santa Clara, CA) or pcDNA3.1 (Invitrogen) vectors. The REG4 constructs were transiently transfected into PC3_shADAM9_ using lipofectamine 2000 (Invitrogen) following the standard protocol. Overexpressed REG4 was confirmed by immunoblotting (R&D System). Secreted REG4 in the conditioned medium was collected and concentrated using centricon YM-3 centrifuge filters (Millipore).

### Ethics Statement

The animal studies were carried out in strict accordance with the recommendations in the Guide for the Care and Use of Laboratory Animals of the National Institutes of Health. The protocol was approved by the Instituional guidelines and an Animal Research Committee of China Medical University was followed for the mouse studies (IACUC# 99-68-N). All surgery was performed under Zoletil (tiletamine-zolazepam) at a dose of 20 mg/kg anesthesia by intraperitoneal injection, and bioluminescence imaging was taken under control by Isoflurance anesthesia. All efforts were made to minimize suffering.

### Statistical Analysis

All data from the *in vitro* studies are presented as the mean ± SD. Differences between groups were analyzed using the two-tailed Student’s *t-test* or as indicated in the figure legend. A *p* value of less than 0.05 was considered to be significant. In the mouse studies, the Mann-Whitney rank-sum test was used for analysis.

## Results

### Knockdown of ADAM9 Expression Decreased Prostate Cancer Cell Proliferation

**Figure 1:**
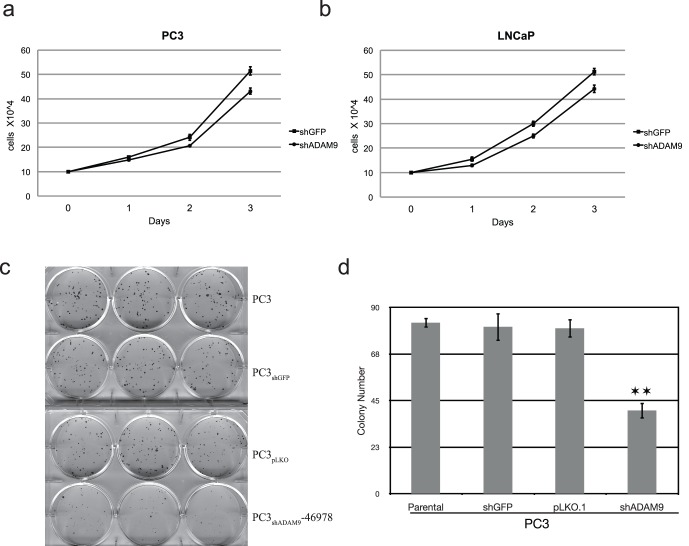
Knockdown of ADAM9 expression decreased prostate cancer cell proliferation. Cell proliferation decreased starting 2 days after seeding (1×10^4^ cells/well) of ADAM9-knockdown PC3 (a) and LNCaP (b) (* Student’s t-test, *p*≤0.05). (c) Clonogenic cell proliferation assays of PC3, PC3_shGFP_, PC3_pLKO_ and PC3_shADAM9_-46978 revealed decreased colonies in PC3_shADAM9_-46978. (d) Quatitative analyses of clonogenic studies demonstrated that the number of cell colonies was lower in lentiviral knockdown of ADAM9 expression compared to wild-type, mock infected, and non-target controls (** Student’s t-test, *p*≤0.001).

To validate the role of ADAM9 in prostate cancer progression, we stably downregulated ADAM9 expression in androgen-independent, bone metastatic PC3 prostate cancer cells and androgen-dependent LNCaP prostate cancer cells with either retroviral or lentiviral vectors carrying ADAM9-specific shRNA. The resultant ADAM9 knockdown cell lines PC3_shADAM9_ and LNCaP_shADAM9_, which showed an approximately 88% and 76% reduction respectively in ADAM9 protein expression when compared with either empty vector (pSM2C retroviral vector) or control shRNA (lentiviral-shGFP) (Fig. S2), were selected for the following study. A 3-day short-term cell proliferation assay indicated that both PC3_shADAM9_ (Fig. 1a) and LNCaP_shADAM9_ (Fig. 1b) cells exhibited a significant decrease in cellular proliferation. This delayed growth of ADAM9 knockdown cells was further confirmed by clonogenic assays in which a 40–50% reduction in PC3_shADAM9_ cells was seen after 2 weeks of cell culture (Figs. 1c and 1d).

**Figure 2:**
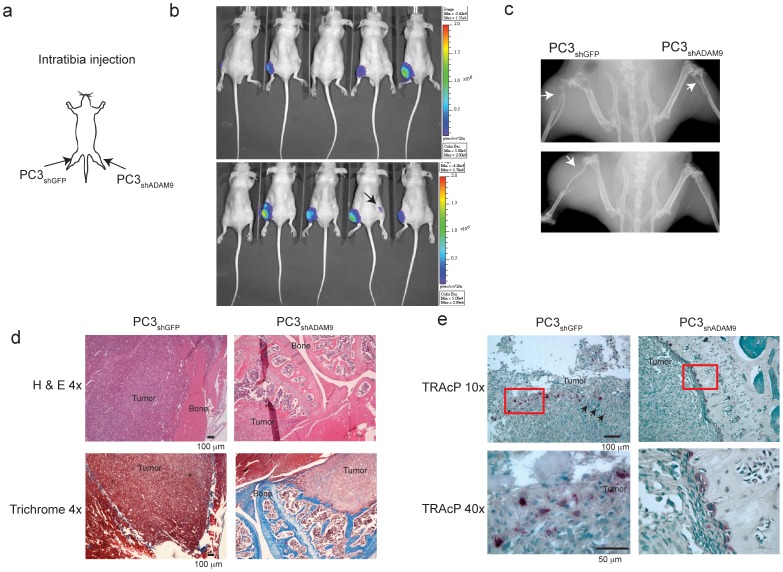
ADAM9 knockdown reduces PC3-induced tumor growth and osteolytic activity. (a) PC3_shGFP_ and PC3_shADAM9_ were injected into the left and right tibia of the same mouse (n = 10). (b) Bioluminescence imaging at 40 days post-inoculation. Black arrows show the only signal detected in a PC3_shADAM9_ bone lesion. (c) Computerized radiographic scanning of lesion area. Upper image: Bone destruction is extensive in the left leg compared to the right leg; Lower image: mouse with a destructive bone lesion in the left leg and no lesion in the right leg. (d) Histological imaging showing that both trabecular and cortical bone was replaced by the PC3_shGFP_ tumor. In contrast, trabecular and cortical bone was still intact in the PC3_shADAM9_ tumor (H & E). Masson’s trichrome staining indicated only traces of bone left in PC3_shGFP_ compared to PC3_shADAM9_ tumors (Trichrome). (e) TRAcP analysis of osteoclast activity. Arrow indicates osteoclasts observed in a PC3_shGFP_ tumor. Red highlight indicates loci of 40× enlarge.

To assess the growth difference between ADAM9-proficient and ADAM9-deficient cancer cells in bone, the primary metastatic site for prostate cancer, luciferase-tagged PC3_shGFP_ and PC3_shADAM9_ were intratibially injected into the same nude mouse but at different legs (Fig. 2a, n = 10). The collective bioluminescent imaging data showed that the incidence of PC3_shGFP_ tumor development was 80%, and the tumors were detectable as early as 15 days post-injection. In contrast, only 10% of mice tibias injected with PC3_shADAM9_ had detectable tumor growth. The bioluminescence intensity was approximately one log lower than most of the PC3_shGFP_ tumors at 40 days post-injection (Fig. 2b). A similar result was also obtained with subcutaneous xenografts (Fig. S3), which indicated an important role of ADAM9 in prostate cancer cell proliferation and tumor growth.

### ADAM9 Gene Silencing Decreased in vivo Osteolytic Bone Lesions Caused by PC3 Tumors in Mice

Since, PC3 cells are known to induce osteolytic lesions *in vivo*, we determined whether knockdown of ADAM9 expression could attenuate tumor-induced bone resorption. Computerized radiographic scanning of the affected legs revealed severe osteolytic bone defects in the left tibia (injected with PC3_shGFP_), whereas the mass appeared normal in the right tibia (injected with PC3_shADAM9_) (Fig. 2c). Masson’s trichrome staining of left leg confirmed that both trabecular and cortical bone in the proximal tibial metaphysis had been destroyed, and the marrow cavity had been replaced by tumors. In contrast, the trabecular and cortical bones remained intact in PC3_shADAM9_-injected right tibia and, except for one incident in which weak bioluminescence signals had comparatively small tumors in the marrow space with mild bone destruction (Fig. 2d). Additionally, the number and extent of mature osteoclasts in the bone-tumor interface of PC3_shGFP_-injected tibias were much higher than those of PC3_shADAM9_-injected tibias, as determined by tartrate-resistant acid phosphatase (TRAcP) staining (*p*<0.01) (Fig. 2e), indicating that silencing the expression of ADAM9 reduced the ability of prostate cancer cells to induce osteoclastogenesis in the bone.

### Targeting ADAM9 Expression by Intratumoral Delivery of shRNA Suppresses Prostate Cancer Growth in Mice

**Figure 3:**
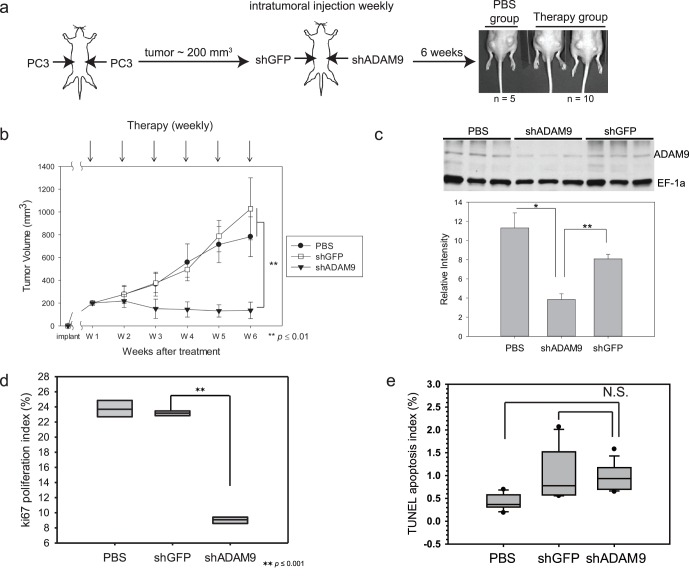
ADAM9 reduction therapy decreased tumor volume by inhibiting cancer cell proliferation. (a) Schematic of ADAM9 knockdown therapy experiment. Mice were injected subcutaneously with PC3 cells in both flanks, and tumor size was measured twice a week until the size reached approximately 200 mm^3^. Mice then received injections of either PBS into both tumor sites (n = 5) or shGFP lentivirus into the left side tumor and shADAM9 into the right side (n = 10). Viruses were injected and tumors measured weekly for 6 consecutive weeks. (b) After shADAM9 therapy, tumor volumes did not increase compared to shGFP therapy or PBS controls (** *p*≤0.01, Student’s *t* test). (c) Immunoblotting assay of ADAM9 expression confirms decreased ADAM9 expression after shADAM9 therapy. EF-1α was used as a loading control (* *p*≤0.05; ** *p*≤0.01, Student’s *t* test). (d) Statistical analysis of ki67 staining. shADAM9 therapy significantly decreased cell proliferation activities in PC3 tumors. PBS and shGFP therapy showed no difference in the proliferation index (** *p*≤0.001, One Way ANOVA). (e) Statistical analysis of TUNEL staining showed no significant (N.S.) difference between therapies (*p*≥0.05, One Way ANOVA).

To examine whether shRNA-mediated inhibition of ADAM9 expression represents a promising strategy for prostate cancer gene therapy, PC3 cells were subcutaneously inoculated into both flanks of nude mice. The tumors were allowed to grow to a size of approximately 200 mm^3^. Ten of fifteen mice that had PC3 tumors on both flanks were selected to receive the intra-tumoral injection of 1×10^5^ cfu lentiviral vectors (LV) carrying shGFP (left site flank) or shADAM9 (right site flank) weekly for a total of 6 weeks (Fig. 3a), and the remaining 5 mice served as controls by receiving PBS injections. Decreased prostate tumor growth was observed in tumors treated with LV-shADAM9 compared to those treated with LV-shGFP or PBS (Fig. 3b). The average tumor size after 6 weeks of therapy with PBS or LV-shGFP was 782.7±174.6 mm^3^ and 1027±272.2 mm^3^, respectively. By contrast, the size of tumors treated with LV-shADAM9 therapy was 135.9±72.4 mm^3^. The level of ADAM9 expression was decreased in tumors treated with LV-shADAM9 therapy, as confirmed by both tissue staining (Fig. S4d–f) and immunoblotting (Fig. 3c; *p*≤0.05).

To determine the cellular responses associated with LV-shADAM9 therapy, tumor proliferation (Ki-67 index, Fig. S4g–i) and apoptosis marker staining (TUNEL index, Fig. S4j–l) of tumor sections were conducted. We did not find a significant difference between the number of apoptotic cells in controls and LV-shADAM9 (Fig. 3e and Fig. S4j–l). In contrast, the treatment with LV-shADAM9 resulted in a dramatic decrease in the number of Ki-67-positive cells compared to both PBS and LV-shGFP treatments (Fig. 3d and Fig. S4g–i). These data demonstrated that *in vivo* delivery of LV-shADAM9 effectively regresses tumor growth by inhibition of cell proliferation rather than induction of apoptotic cell death.

### Knockdown ADAM9 Induces Cell Cycle Arrest at the G1/S Transition Under Stress Conditions

**Figure 4:**
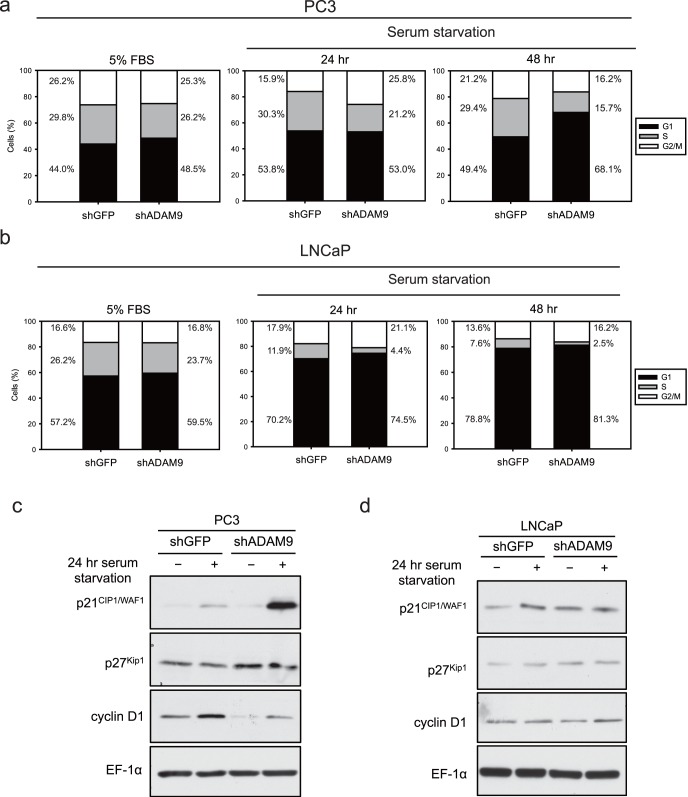
ADAM9 silencing induced G_1_ arrest. Starvation reduced the S-phase cell population in ADAM9 knockdown cancer cells compared to that of controls in PC3 (a), LNCaP (b). (c) Expression levels of p21^Cip1/WAF1^, p27^Kip1^, and cyclin D1 increased in shADAM9 cells under starvation stress conditions in PC3 cells. (d) Expression levels of p21^Cip1/WAF1^ and p27^Kip1^ increased in LNCaP_shADAM9_ under both normal and starvation conditions.

To further delineate the mechanism of LV-shADAM9 therapy-induced tumor growth inhibition, the cell cycle distribution between ADAM9-proficient PC3_shGFP_ and ADAM9-deficient PC3_shADAM9_ cells cultured under serum-starvation or serum-supplemented (5% FBS) conditions were analyzed. While PC3_shGFP_ and PC3_shADAM9_, under normal conditions, did not show a change in the cell cycle profiles, the S-phase population was significantly decreased with time (from 24 to 48 hr) in serum-starved PC3_shADAM9_ cells as compared with that in PC3_shGFP_. This reduction was accompanied by the increased percentage of cells in the G1 phase (Fig. 4a). A similar result was obtained by using LNCaP cells (Fig. 4b). Moreover, immunoblotting studies of G1/S transition-related proteins revealed a significantly higher level of p21^Cip1/WAF1^. Furthermore, a lower level of cyclin D1 was induced in PC3_shADAM9_ than in PC3_shGFP_ under serum starvation conditions (Fig. 4c). Although serum starvation did not cause any further changes in the expression level of p27^Kip1^ in either PC3_shADAM9_ or PC3_shGFP_, the basal level of p27^Kip1^ was higher in PC3_shADAM9_. Taken together, these results indicate that the exposure of PC3_shADAM9_ to serum starvation elicits a G1 cell cycle arrest. Increased expressions of p21^Cip1/WAF1^and p27^Kip1^ were also detected in LNCaP_shADAM9_ under normal culture conditions (Fig. 4d).

**Figure 5:**
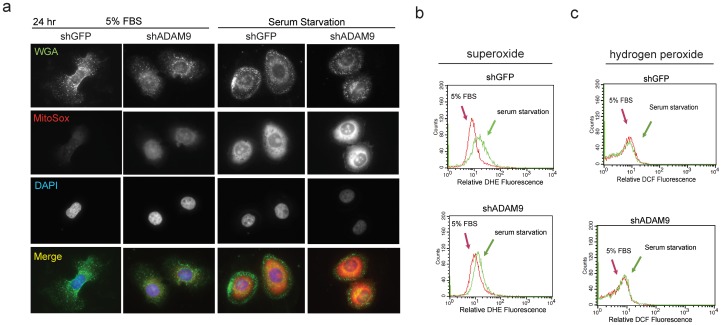
Knockdown ADAM9 expression enhances endogenous superoxide levels under stress condition. (a) PC3_shGFP_ and PC3_shADAM9_ cells were cultured in either 5% FBS or under serum starvation for 24 hours, followed by treatment with 0.5 µM MitoSOX fluorescence. Equal intensity of fluorescence was used in each image capture for quantification of superoxide levels. (b) Elevation of endogenous superoxide concentrations level can be detected during serum starvation in both PC3_shGFP_ and PC3_shADAM9_. (c) Endogenous hydrogen peroxide levels did not differ between cells cultured in 5% FBS and under serum starvation.

Owing to our previous finding that ADAM9 is a stress-responsive protein that may support prostate cancer cell survival and progression under intense oxidative stress conditions [13], [19], we sought to determine whether serum starvation, an *in vitro* condition that mimics the hostile tumor microenvironment [16], [19], can induce the intracellular oxidative stress in which ADAM9-deficient cells failed to overcome. We detected an increase in endogenous superoxide levels in both PC3_shGFP_ and PC3_shADAM9_ cells after 24 hours of serum starvation as compared to cells grown under serum supplemented conditions as measured by live cell imaging with a MitoSox red fluorescent probe (Fig. 5a) as well as flow cytometry with the fluorescent dye, dihydroethidium (DHE) (Fig. 5b). Notably, PC3_shADAM9_ exhibited significantly higher superoxide content than PC3_shGFP_, either at the basal level or induced by serum starvation. In contrast, no changes in the level of intracellular hydrogen peroxide were found in either PC3_shGFP_ or PC3_shADAM9_ cells, as assayed by flow cytometry with DCFH-DA probes (Fig. 5c). These data demonstrate that serum starvation-induced oxidative stress in prostate cancer cells is mediated, at least in part, through superoxide but not hydrogen peroxide, a result similar to our previous observation involving ionizing radiation-induced reactive oxygen species (ROS) generation [16].

### Knockdown of ADAM9 Expression Results in the Inhibition of REG4 Dependent Cell Cycle Regulation

**Figure 6:**
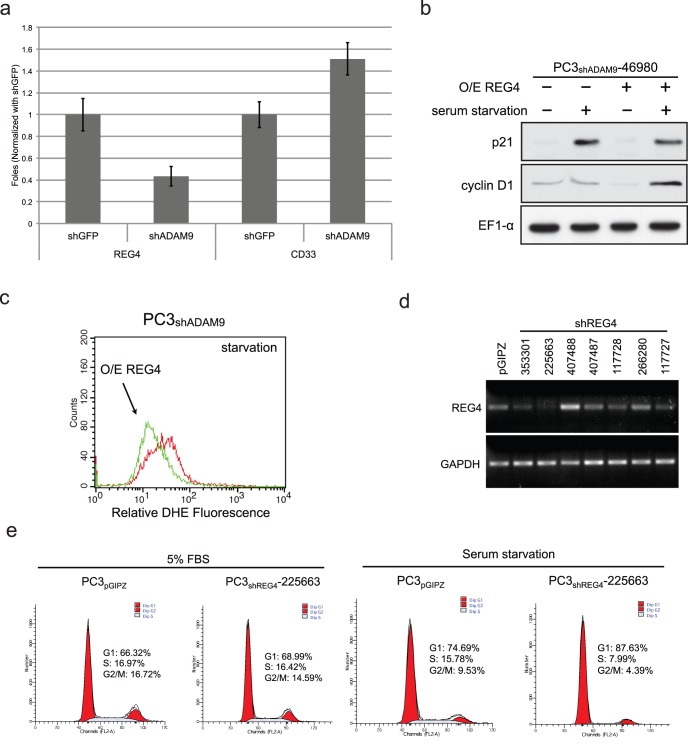
Decreased REG4 and increased p21^Cip1/WAF1^, p27^Kip1^, and cyclin D1 expression in ADAM9 knockdown prostate cancer cells. (a) Quantitative PCR confirmed the decrease of REG4 mRNA and increase of CD33 mRNA. (b) Overexpression of REG4 decreased p21^Cip1/WAF1^ expression under FBS starvation conditions in PC3_shADAM9_. (c) The starvation-induced increase in superoxide levels was reversed by overexpression of REG4 in PC3_shADAM9_. (d) The strongest inhibition of REG4 expression by shREG4 lentiviral vectorwas observed in PC3_shREG4_-225663 by RT-PCR. (e) PC3_shREG4_-225663 reduced the S-phase and increased the G1-phase populations under starvation conditions.

To further determine the underlying mechanism for shADAM9-induced G1 phase growth arrest under stress conditions, cDNA microarray analysis of differential gene expression was conducted in PC3_shADAM9_ cells. To exclude any off-target effects of shRNA on ADAM9 driven genes, two sets of PC3_shADAM9_ cell line, PC3_shADAM9_-17130 and PC3_shADAM9_-46980, which were established with two distinct shRNA constructs by a retroviral system and a lentiviral system (Fig. S1). The corresponding control cell lines were also used in this assay. Intersection analyses revealed an increase in CD33 and a decrease in regenerating islet-derived family member 4 (REG4), insulin-like growth factor binding protein 3 (IGFBP3) and ADAM9 mRNA in these two PC3_shADAM9_ cell lines (Fig. S5a). The transcriptional alterations of REG4 and CD33 in PC3_shADAM9_ cell lines were further confirmed by RT-PCR (Fig. S5b) and quantitative PCR (Fig. 6a). A recent study demonstrated that knockdown of REG4 expression resulted in the upregulation of p21^Cip1/WAF1^ and p27^Kip1^ [20]. We, therefore, wanted to determine whether the upregulation of p21^Cip1/WAF1^ in PC3_shADAM9_ under stress conditions (Fig. 4) is mediated by the downregulation of REG4 gene function. Hence, we transiently overexpressed REG4 in PC3_shADAM9_ cells (Fig. S5c). While overexpression of REG4 has no effects on the basal level expression of p21^Cip1/WAF1^ and cyclin D1 in PC3_shADAM9_, serum starvation-induced p21^Cip1/WAF1^ expression was decreased and cyclin D1 was increased as compared to vector control cells upon serum starvation (Fig. 6b). This change in cell cycle-related protein expression in REG4-overexpressed PC3_shADAM9_ cells under serum starvation was associated with decreased endogenous superoxide levels (Fig. 6c). Conversely, the knockdown of REG4 in PC3 cells using a sequence-specific shRNA against REG4 (Fig. 6d) caused a striking decrease of cells in the S phase from 16.42% to 7.99% accompanied by an increase in the G1 phase from 68.99% to 87.63% after 24 h serum starvation (Fig. 6e). There was no significant difference in the cell cycle distribution of vector control PC3 (PC3_pGIPZ_) cells between normal culture and serum-starved condition. Taken together, these data demonstrated that shADAM9 mediated stress-induced G1 arrest is due, in part, to the downregulation of REG4 expression and the associated changes in the downstream effector, p21^Cip1/WAF1^.

## Discussion

Androgen-deprivation therapy is generally the initial treatment for men with advanced prostate cancer. Although patients have high response rates to the initial hormone therapy, nearly all of them eventually develop progressive and metastatic castrate-resistant disease. Docetaxel in combination with prednisone is now regarded as the standard first-line chemotherapy in patients with symptomatic castration-resistant prostate cancer (CRPC) [21]. However, therapeutic responses are generally not sustained and CRPC remains an invariably fatal disease. The mortality of combination therapies only provided an improved median survival benefit of 2 to 3 months compared to monotherapies [5], [6], [21], [22]. For this reason, treatment of patients with CRPC remains a significant clinical challenge. In addition, the major impact on the quality of life of most patients with CRPC occurs as a result of their disease metastasizing to bone. The current palliative treatment options for patients with CRPC include radiotherapy, inhibitors of androgen biosynthesis such as abiraterone acetate [23] and MDV3100 [24], bone-targeted therapy using biphosphonate [25] or denosumab [26], and vaccine-based immunotherapy such as Sipuleucel-T [27], GVAX [28], and PROSTVAC-VF [29]. Although denosumab therapy provides a median bone-free metastasis survival of 6 months, CRPC patients maintain bone metastasis at later cancer stages [30]. Recent studies demonstrated that cancer cells treated with either radiation therapy or chemotherapy agents, such as docetaxel, can enhance the level of ROS [31]. Maintaining lower concentration of endogenous ROS in docetaxel-resistant cancer cells correlates with cell survival during docetaxel therapy [32]. Therefore, it is urgent to enhance therapeutic effects by decreasing drug resistance of malignant cancer cells.

In the present study, we demonstrated the ability of ADAM9 shRNA impaired androgen-independent prostate cancer PC3 tumor formation and cancer-induced osteolysis in a skeletal metastasis xenograft model. We also presented data indicating the feasibility of using *in vivo* lentivirus-delivered ADAM9 shRNA to reduce the tumor burden in animals. Taken together this research may provide an additional targeted approach for not only improving overall survival rates in men with CRPC but also providing clinical benefits for rapid relief of osteolysis induced complication. While ADAM9 has been implicated in tumor progression and prognosis of a variety of cancers, due, in part, to the role of metalloproteinases in cell migration and invasion [10], [33]–[35], to date cell cycle analyses and modulation of cell cycle proteins have not been investigated in the context of ADAM9. We also show, for the first time that ADAM9 knockdown induces cell cycle arrest at the G1/S transition in prostate cancer cells under serum starvation conditions. This is due, in part, to the induction of oxidative stress, with an associated upregulation of p21^Cip1/WAF1^ and p27^Kip1^ and the inhibition of cyclin D1. The mechanism of p21^Cip1/WAF1^ and p27^Kip1^ induction is through, at least in part, decreased REG4 gene expression. Combined with our previous findings that oxidative stress induces ADAM9 expression in prostate cancer cells [13], and knockdown of ADAM9 sensitizes prostate cancer cells to ionizing irradiation and chemotherapeutic agents including doxorubicin, cisplatin, taxotere, gemcitabine, and etoposide [16], these results reveal a novel regulatory pathway of this oncoprotein in protecting cancer cells against oxidative damage and other stressors in the prostate tumor microenvironment. Alkylating agents such as doxorubicin, cisplatin, and etoposide as well as radioactive pharmaceutics are well known cytotoxic agents that function through the regulation of oxidative stress [7], [36]. Therefore, therapeutic stresses, which can induce endogenous oxidative responses and survival pathways by activating cyclin D1 and inactivating p21^Cip1/WAF1^ and p27^Kip1^, may inhibit therapeutic induced apoptosis [37]. We also observed an increase in superoxide but not hydrogen peroxide in radiation therapy [16]. Knockdown of ADAM9 expression enhanced the sensitivity of cancer cells to radiation (Fig. S6). Therefore, our current results provide a rationale for the application of ADAM9 targeting strategy, LV-ADAM9 shRNA for example, as a potential adjuvant modality in combination with currently available redox-active therapeutic agents for the management of CRPC.

**Figure 7:**
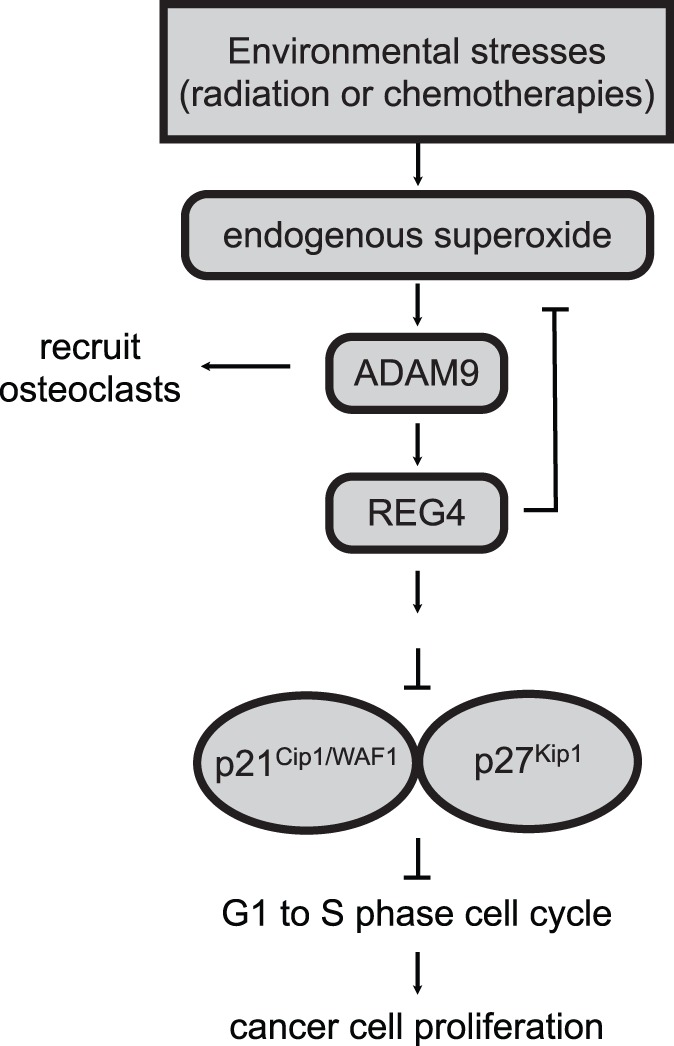
Scheme demonstrated the role of ADAM9 in the regulation of REG4 and p21^Cip1/WAF1^/p27^Kip1^ activities in prostate cancer cells during environmental stresses, such as radiation therapy or chemotherapy.

The regenerating islet-derived (REG) family is a group of small secretory proteins that belong to the C-type lectin superfamily. The physiological function of REG family is mainly involved in proliferation, differentiation and inflammation of cells in the digestive system [38]. Within this family, REG4 has been reported to be associated with human gastrointestinal malignancies [39]–[42] and prostate cancer [43]–[45]. Although upregulated REG4 is frequently observed in CRPC [43], [44] and is considered to be a prognostic indicator of cancer relapse after radical prostatectomy [45], the biological function of REG4 in prostate cancer progression has only begun to be explored. Several lines of studies in other cancer types have demonstrated an impact of REG4 on tumor growth, invasion, metastasis, and resistance to apoptosis [20], [46], [47], which is similar to the role of ADAM9 in prostate cancer seen in the previous and the present studies. Given that knockdown ADAM9 decreases the expression of REG4 (Fig. 6), we suggest a regulatory role of ADAM9 in REG4-mediated prostate cancer progression (Fig. 7). In a colon cancer model, REG4 was induced by growth factors, including transforming growth factor-alpha, epidermal growth factor (EGF), basic fibroblast growth factor and hepatocyte growth factor [48]. Since ADAM9 is involved in ectodomain shedding of heparin binding EGF-like factor (HB-EGF) and subsequent activation of EGFR [8], [10], it is tempting to hypothesize that ADAM9 induces REG4 expression through the transaction of EGFR by cleaved HB-EGF. Further investigation on the connection between ADAM9 and REG4 signlaing in cell proliferation and cell survival will improve the understanding of the underlying mechanisms in CRPC progression and therapy resistance.

In conclusion, the findings presented here thus establish that ADAM9 is essential for cancer progression during the transition to malignancy. Loss of ADAM9 in prostate cancer cells results in the impairment of proliferation and the osteolytic reaction, as well as causing G_1_/G_0_ cell cycle arrest. We identified the G_1_-to-S phase negative regulators p21^Cip1/WAF1^and p27^Kip1^ as downstream targets of the ADAM9-REG4 signaling pathway. In a tumor microenvironment, this pathway couples the stress-induced production of ROS to proliferation, G_1_ phase arrest, and cellular senescence induced by combination therapies.

## Supporting Information

Figure S1 **Scheme illustration of retroviral (up) and lentiviral (bottom) shRNA targets to ADAM9 mRNA and lentiviral shRNA targets to Reg4 mRNA.** Targeted sequences were listed.
(PDF)

Figure S2 (a) Immunoblotting validated ADAM9 expression and revealed the knockdown of ADAM9 by Retroviral shADAM9-17130. Vector, pSM2C, were also transfected into PC3 and used as negative control. To select knockdown clone, several clones were also examined. Clone 7 was selected and used in most of our studies. (b) Immunoblotting confirmed knockdown effects of ADAM9 gene in PC3 using lentivral knockdown systems. 46978 and 46980 were selected and shGFP were used as control studies. (c) Immunoblotting confirmed the knockdown of ADAM9 in LNCaP.
(PDF)

Figure S3 **Inhibition of PC3 prostate cancer cell growth in mice after knockdown ADAM9 expression.** (a) Scheme illustration of subcutaneous inoculation of PC3_pSM2C_ and PC3_shADAM9_ in same mice. PC3_pSM2C_ was implanted in the left size and PC3_shADAM9_ in the right site of lower back of same mice. (b) Bioluminescence imaging obtained at day-30 post inoculation. Only two mice revealed a weak bioluminescence signal on the right site of PC3_shADAM9_ (black arrow). (c) On day 15, signal was first observed at the PC3_pSM2C_ implant site in 50% of the mice, increasing to 90% on day 25. The number of mice with a signal at the PC3_shADAM9_ implant site remained at 10% (mice, n = 20; ** p≤0.001). (d) Tumor volume was measured every 5 days starting at day 15 and ending at day 60. Tumor volume was 281±91 mm^3^ in PC3_pSM2C_ and 140±37 mm^3^ in PC3_shADAM9_ on day 60 (means ± SD) (*p≤0.05 one-way ANOVA).
(PDF)

Figure S4 **Histopathology of tumors after therapies revealed reduction of ADAM9 and Ki-67 staining in shADAM9 group.** H & E staining of tumor region treated either with PBS, shGFP lentivirus or shADAM9 lentivirus therapies (a-c). ADAM9 immunohistochemistry staining showed positive of ADAM9 in PBS and shGFP therapies (d and e). (f) By contrast, decreased ADAM9 positive expression confirmed the knockdown of ADAM9 in the shADAM9 therapy group. Proliferation index of shADAM9 therapy indicated decreased of Ki-67 positive staining in shADAM9 group (i) compared to PBS or shGFP therapy groups (g and h). Apoptosis index showed no different in all therapeutic studies (j–l).
(PDF)

Figure S5 **Intersection microarray analyses revealed the increased expression of CD33 and decreased of REG4 expression after knockdown of ADAM9 expression.** (a) Two microarrays and intersection analysis of common gene changes between retrovirus and lentivirus shADAM9 profiling. Results indicate that mRNA levels of CD33 increased and those of REG4, IGFBP3, and ADAM9 decreased. (b) Confirmation of the decrease of REG4 mRNA and increase of CD33 mRNA. (c) Immunoblot analysis confirmed the overexpression of REG4 expression that secreted in the concentrated conditioned medium.
(PDF)

Figure S6 **Silencing ADAM9 enhanced radiotherapy efficacy.** (a) Imaging of clonogenic analysis at 0, 10, 15, 20 and 25 Gy of radiation. PC3, PC3_shGFP_, PC3_pLKO.1_ and PC3_shADAM9_ cells were plated 100 cells/well and exposed to radiation the next day. Colony was measured 14-day after plating. (b) Statistic analyses of cologenic study of (a) that showed significantly enhance radiation sensitivities after knockdown of ADAM9 expression. * *p*≤0.05, Student’s *t* test.
(PDF)

Materials and Methods S1 (DOCX)
