# Supplementary material for: In Vivo Targeting of ADAM9 Gene Expression Using Lentivirus-Delivered shRNA Suppresses Prostate Cancer Growth by Regulating REG4 Dependent Cell Cycle Progression
Source: PLoS One. 2013 Jan 16;8(1):e53795. doi: 10.1371/journal.pone.0053795 (PMC3547060; doi:10.1371/journal.pone.0053795)
Supplement: Figure S1 — Scheme illustration of retroviral (up) and lentiviral (bottom) shRNA targets to ADAM9 mRNA and lentiviral shRNA targets to Reg4 mRNA. Targeted sequences were listed. (PDF) [file pone.0053795.s001.pdf]

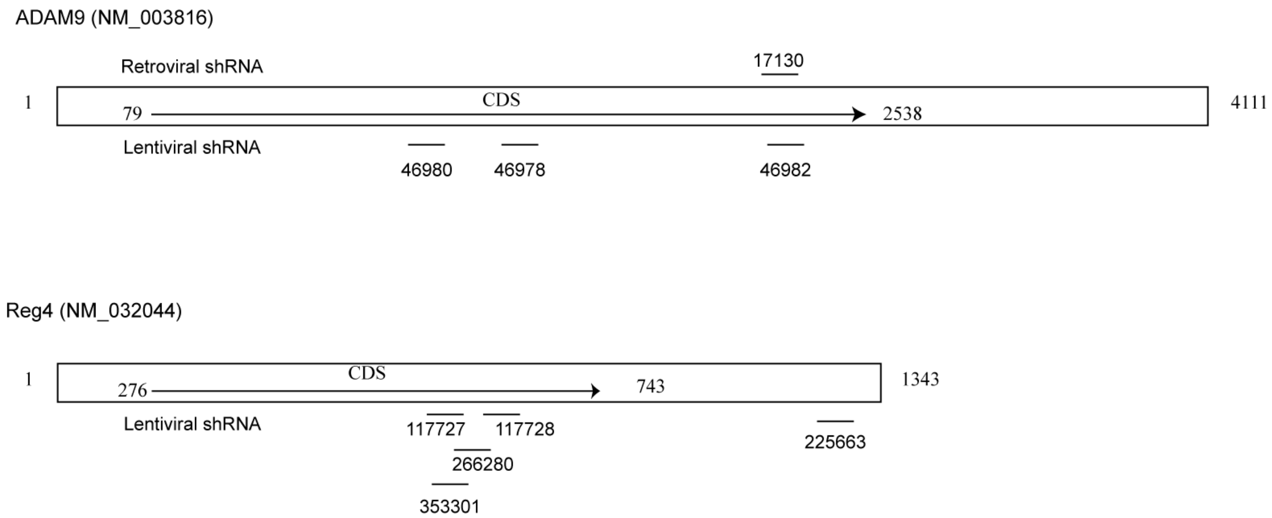

shADAM9 target sequences:
17130: 5'-CTCCCAGAGAAGTTCCTAT-3' (2363-2381)
46978: 5'-GCCAGTATTATGATGCTCAAT-3' (1628-1648)
46980: 5'-GCCAGAATAACAAAGCCTATT-3' (1592-1612)
46982: 5'-CCCAGAGAAGTTCCTATATAT-3' (2365-2385)

shReg4 target sequences:
117727: 5'-GAGTGTCAGTCTTACGGAA-3' (435-453)
117728: 5'-CAGAGTACATAAGTGGCTA-3' (505-523)
225663: 5'-GGCCATACATTCCTTTAAT-3' (1238-1256)
266280: 5'-CACCATAGCAGAGTACATA-3' (497-515)
353301: 5'-GGCATCTATCCTGAGTTTA-3' (467-485)

Supplement Figure S1. Scheme illustration of retroviral (up) and lentiviral (bottom) shRNA targets to ADAM9 mRNA and lentiviral shRNA targets to Reg4 mRNA. Targeted sequences were listed.
