# Supplementary material for: In Vivo Targeting of ADAM9 Gene Expression Using Lentivirus-Delivered shRNA Suppresses Prostate Cancer Growth by Regulating REG4 Dependent Cell Cycle Progression
Source: PLoS One. 2013 Jan 16;8(1):e53795. doi: 10.1371/journal.pone.0053795 (PMC3547060; doi:10.1371/journal.pone.0053795)
Supplement: Figure S2 — (a) Immunoblotting validated ADAM9 expression and revealed the knockdown of ADAM9 by Retroviral shADAM9-17130. Vector, pSM2C, were also transfected into PC3 and used as negative control. To select knockdown clone, several clones were also examined. Clone 7 was selected and used in most of our studies. (b) Immunoblotting confirmed knockdown effects of ADAM9 gene in PC3 using lentivral knockdown systems. 46978 and 46980 were selected and shGFP were used as control studies. (c) Immunoblotting confirmed the knockdown of ADAM9 in LNCaP. (PDF) [file pone.0053795.s002.pdf]

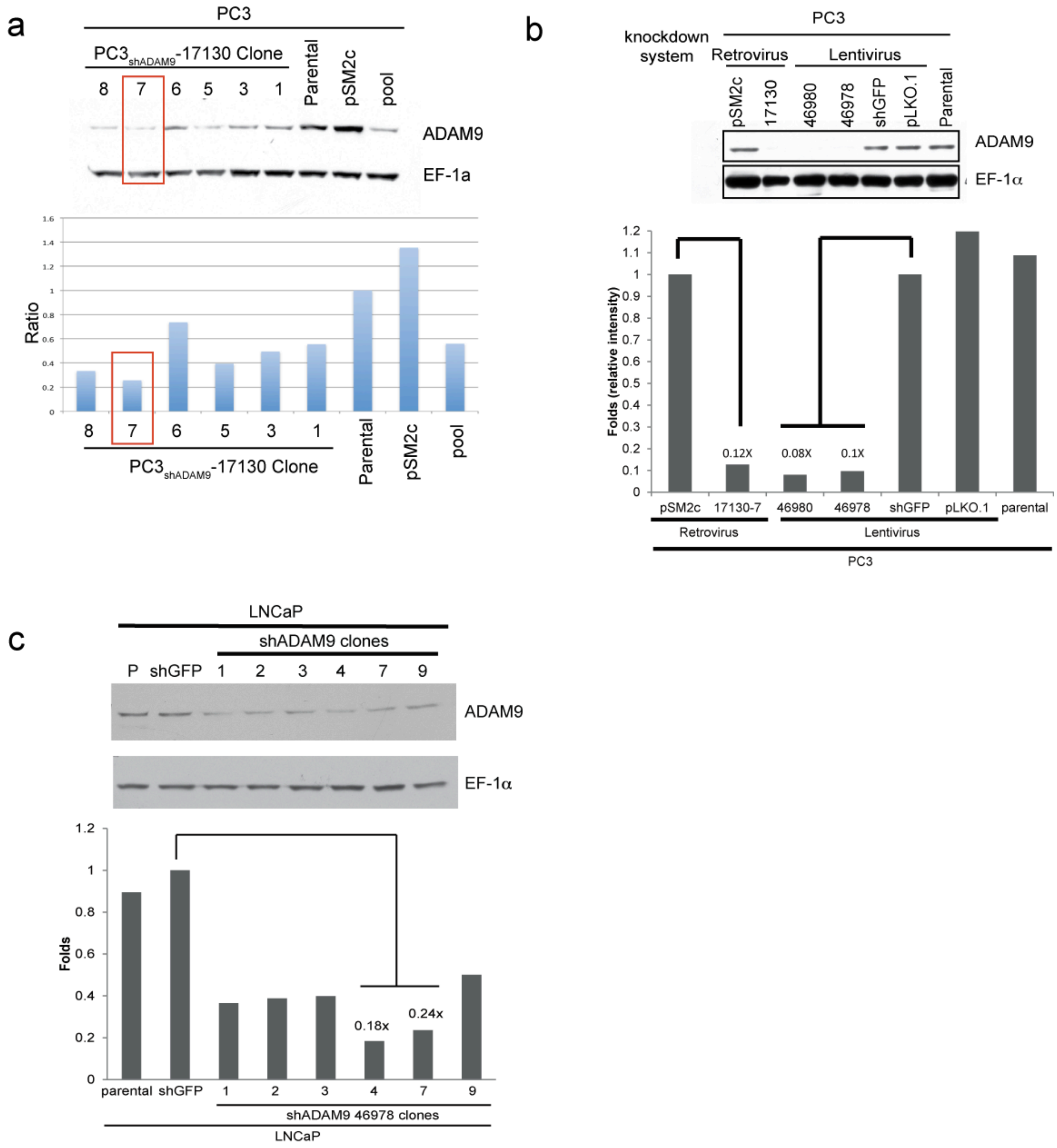

Supplement Figure S2. (a) Immunoblotting validated ADAM9 expression and revealed the knockdown of ADAM9 by Retroviral shADAM9-17130. Vector, pSM2C, were also transfected into PC3 and used as negative control. To select knockdown clone, several clones were also examined. Clone 7 was selected and used in most of our studies. (b) Immunoblotting confirmed knockdown effects of ADAM9 gene in PC3 using lentivral knockdown systems. 46978 and 46980 were selected and shGFP were used as control studies. (c) Immunoblotting confirmed the knockdown of ADAM9 in LNCaP.
