# Supplementary material for: In Vivo Targeting of ADAM9 Gene Expression Using Lentivirus-Delivered shRNA Suppresses Prostate Cancer Growth by Regulating REG4 Dependent Cell Cycle Progression
Source: PLoS One. 2013 Jan 16;8(1):e53795. doi: 10.1371/journal.pone.0053795 (PMC3547060; doi:10.1371/journal.pone.0053795)
Supplement: Figure S3 — Inhibition of PC3 prostate cancer cell growth in mice after knockdown ADAM9 expression. (a) Scheme illustration of subcutaneous inoculation of PC3pSM2C and PC3shADAM9 in same mice. PC3pSM2C was implanted in the left size and PC3shADAM9 in the right site of lower back of same mice. (b) Bioluminescence imaging obtained at day-30 post inoculation. Only two mice revealed a weak bioluminescence signal on the right site of PC3shADAM9 (black arrow). (c) On day 15, signal was first observed at the PC3pSM2C implant site in 50% of the mice, increasing to 90% on day 25. The number of mice with a signal at the PC3shADAM9 implant site remained at 10% (mice, n = 20; ** p≤0.001). (d) Tumor volume was measured every 5 days starting at day 15 and ending at day 60. Tumor volume was 281±91 mm3 in PC3pSM2C and 140±37 mm3 in PC3shADAM9 on day 60 (means ± SD) (*p≤0.05 one-way ANOVA). (PDF) [file pone.0053795.s003.pdf]

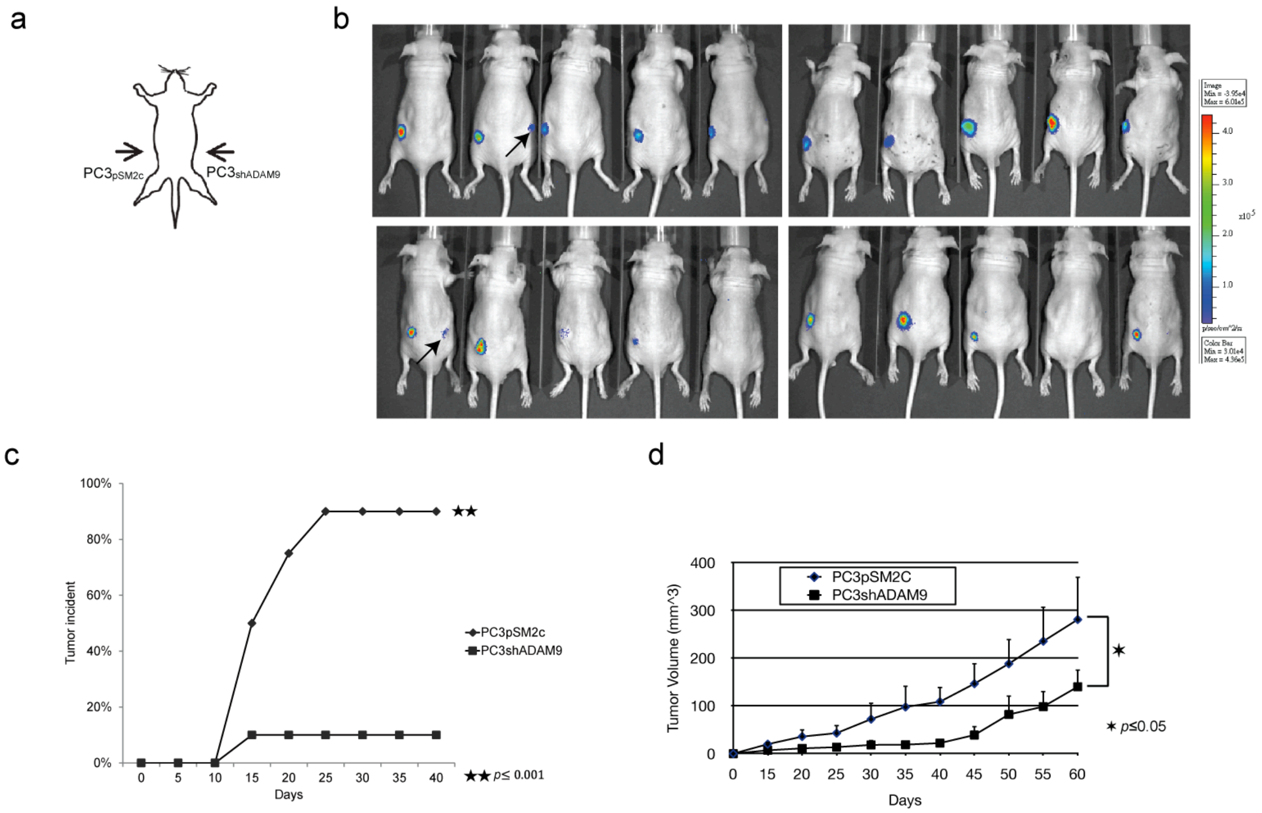

Supplement Figure S3. Inhibition of PC3 prostate cancer cell growth in mice after knockdown ADAM9 expression. (a) Scheme illustration of subcutaneous inoculation of $PC3_{pSM2C}$ and $PC3_{shADAM9}$ in same mice. $PC3_{pSM2C}$ was implanted in the left size and $PC3_{shADAM9}$ in the right site of lower back of same mice. (b) Bioluminescence imaging obtained at day-30 post inoculation. Only two mice revealed a weak bioluminescence signal on the right site of $PC3_{shADAM9}$ (black arrow). (c) On day 15, signal was first observed at the $PC3_{pSM2C}$ implant site in 50% of the mice, increasing to 90% on day 25. The number of mice with a signal at the $PC3_{shADAM9}$ implant site remained at 10% (mice, n=20; ** $p \leq 0.001$). (d) Tumor volume was measured every 5 days starting at day 15 and ending at day 60. Tumor volume was 281 ± 91 mm^3 in $PC3_{pSM2C}$ and 140 ± 37 mm^3 in $PC3_{shADAM9}$ on day 60 (means ± SD) (*$p \leq 0.05$ one-way ANOVA).
