# Supplementary material for: In Vivo Targeting of ADAM9 Gene Expression Using Lentivirus-Delivered shRNA Suppresses Prostate Cancer Growth by Regulating REG4 Dependent Cell Cycle Progression
Source: PLoS One. 2013 Jan 16;8(1):e53795. doi: 10.1371/journal.pone.0053795 (PMC3547060; doi:10.1371/journal.pone.0053795)
Supplement: Figure S4 — Histopathology of tumors after therapies revealed reduction of ADAM9 and Ki-67 staining in shADAM9 group. H & E staining of tumor region treated either with PBS, shGFP lentivirus or shADAM9 lentivirus therapies (a-c). ADAM9 immunohistochemistry staining showed positive of ADAM9 in PBS and shGFP therapies (d and e). (f) By contrast, decreased ADAM9 positive expression confirmed the knockdown of ADAM9 in the shADAM9 therapy group. Proliferation index of shADAM9 therapy indicated decreased of Ki-67 positive staining in shADAM9 group (i) compared to PBS or shGFP therapy groups (g and h). Apoptosis index showed no different in all therapeutic studies (j–l). (PDF) [file pone.0053795.s004.pdf]

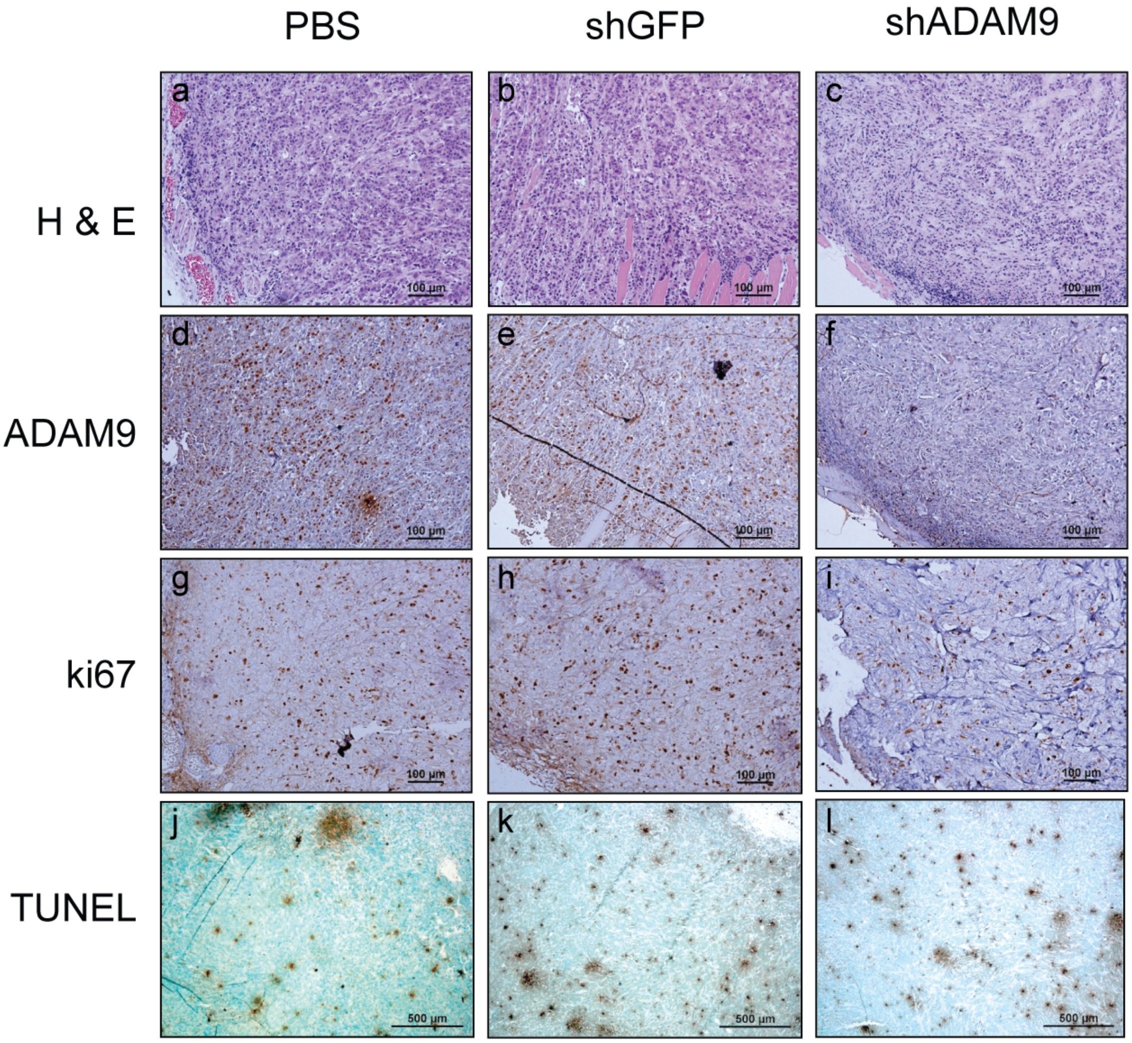

Supplement Figure S4. Histopathology of tumors after therapies revealed reduction of ADAM9 and Ki-67 staining in shADAM9 group. H & E staining of tumor region treated either with PBS, shGFP lentivirus or shADAM9 lentivirus therapies (a-c). ADAM9 immunohistochemistry staining showed positive of ADAM9 in PBS and shGFP therapies (d and e). (f) By contrast, decreased ADAM9 positive expression confirmed the knockdown of ADAM9 in the shADAM9 therapy group. Proliferation index of shADAM9 therapy indicated decreased of Ki-67 positive staining in shADAM9 group (i) compared to PBS or shGFP therapy groups (g and h). Apoptosis index showed no different in all therapeutic studies (j-l).
