# Supplementary material for: In Vivo Targeting of ADAM9 Gene Expression Using Lentivirus-Delivered shRNA Suppresses Prostate Cancer Growth by Regulating REG4 Dependent Cell Cycle Progression
Source: PLoS One. 2013 Jan 16;8(1):e53795. doi: 10.1371/journal.pone.0053795 (PMC3547060; doi:10.1371/journal.pone.0053795)
Supplement: Figure S5 — Intersection microarray analyses revealed the increased expression of CD33 and decreased of REG4 expression after knockdown of ADAM9 expression. (a) Two microarrays and intersection analysis of common gene changes between retrovirus and lentivirus shADAM9 profiling. Results indicate that mRNA levels of CD33 increased and those of REG4, IGFBP3, and ADAM9 decreased. (b) Confirmation of the decrease of REG4 mRNA and increase of CD33 mRNA. (c) Immunoblot analysis confirmed the overexpression of REG4 expression that secreted in the concentrated conditioned medium. (PDF) [file pone.0053795.s005.pdf]

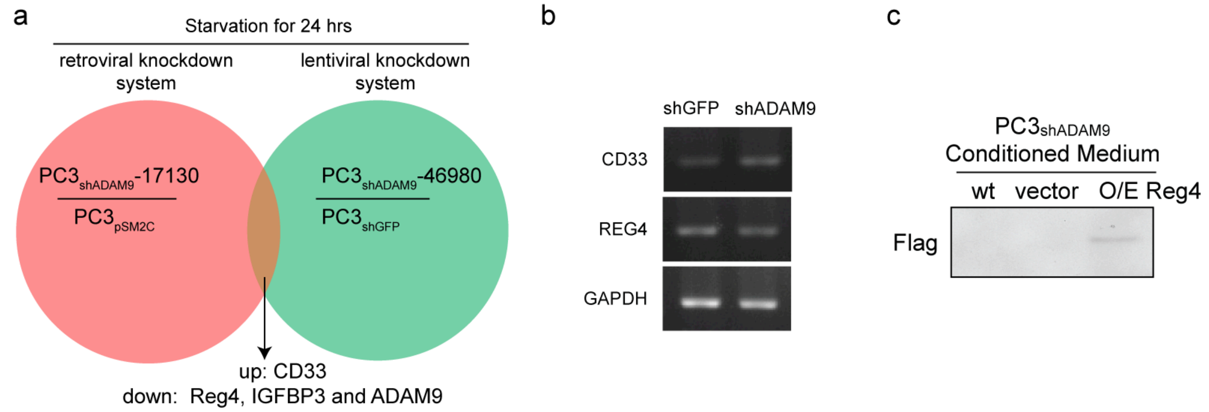

Supplement Figure S5. Intersection microarray analyses revealed the increased expression of CD33 and decreased of REG4 expression after knockdown of ADAM9 expression. (a) Two microarrays and intersection analysis of common gene changes between retrovirus and lentivirus shADAM9 profiling. Results indicate that mRNA levels of CD33 increased and those of REG4, IGFBP3, and ADAM9 decreased. (b) Confirmation of the decrease of REG4 mRNA and increase of CD33 mRNA. (c) Immunoblot analysis confirmed the overexpression of REG4 expression that secreted in the concentrated conditioned medium.
