# Supplementary material for: In Vivo Targeting of ADAM9 Gene Expression Using Lentivirus-Delivered shRNA Suppresses Prostate Cancer Growth by Regulating REG4 Dependent Cell Cycle Progression
Source: PLoS One. 2013 Jan 16;8(1):e53795. doi: 10.1371/journal.pone.0053795 (PMC3547060; doi:10.1371/journal.pone.0053795)
Supplement: Figure S6 — Silencing ADAM9 enhanced radiotherapy efficacy. (a) Imaging of clonogenic analysis at 0, 10, 15, 20 and 25 Gy of radiation. PC3, PC3shGFP, PC3pLKO.1 and PC3shADAM9 cells were plated 100 cells/well and exposed to radiation the next day. Colony was measured 14-day after plating. (b) Statistic analyses of cologenic study of (a) that showed significantly enhance radiation sensitivities after knockdown of ADAM9 expression. * p≤0.05, Student’s t test. (PDF) [file pone.0053795.s006.pdf]

a

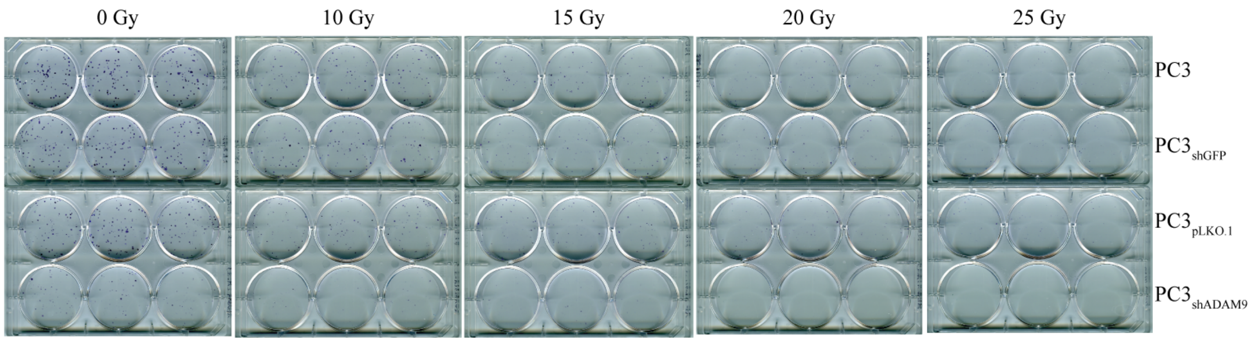

b

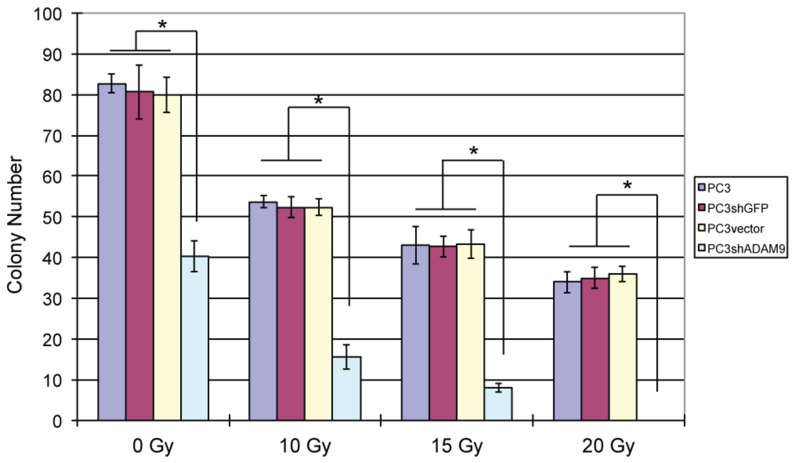

Supplement Figure S6. Silencing ADAM9 enhanced radiotherapy efficacy. (a) Imaging of clonogenic analysis at 0, 10, 15, 20 and 25 Gy of radiation. PC3, $PC3_{shGFP}$, $PC3_{pLKO.1}$ and $PC3_{shADAM9}$ cells were plated 100 cells/well and exposed to radiation the next day. Colony was measured 14-day after plating. (b) Statistic analyses of cologenic study of (a) that showed significantly enhance radiation sensitivities after knockdown of ADAM9 expression. * $p \leq 0.05$, Student's *t* test.
