# Supplementary material for: In Vivo Targeting of ADAM9 Gene Expression Using Lentivirus-Delivered shRNA Suppresses Prostate Cancer Growth by Regulating REG4 Dependent Cell Cycle Progression
Source: PLoS One. 2013 Jan 16;8(1):e53795. doi: 10.1371/journal.pone.0053795 (PMC3547060; doi:10.1371/journal.pone.0053795)
Supplement: Materials and Methods S1 — (DOCX) [file pone.0053795.s007.docx]

Materials and Methods S1

Primers

CD33 forward: 5’-TGTTCCACAGAACCCAACAA-3’

CD33 reverse: 5’-TTCCTCCTGTGGGTCTTCAC-3’

Reg4 forward: 5’-TGCTCCTGGATGGTTTTACC-3’

Reg4 reverse: 5’-TATCGGCTGGCTTCTCTGAT-3’

GAPDH forward: 5’-GAAGCTGAAGGTCGGAGTC-3’

GAPDH reverse: 5’-GAAGATGGTGATGGGATTTC-3’

HindIII-REG4(1-17)F: 5’-AAGCTT-ATGGCTTCCAGAAGCAT-3’

XbaI-REG4(477-459)R: 5’-TCTAGA-CTATGGTCGGTACTTGCAC-3’

*In vivo xenograf models.* Five-week-old male athymic nude (nu/nu) mice obtained from National Laboratory Animal Center at Taiwan were used for subcutaneous (s.c.), intracardiac, and intratibial tumor implantation. Cells were cultured to 100% confluence, trypsinized and enumerated. Mice were sedated with 1.7% isoflurane mixed with air for s.c. tumor implantation. Xenograft tumors were established by s.c. injection of 106 PC3 cells expressing pSM2c or shADAM9 cells in 100 μL of PBS into both sides of each mouse. Animals were sacrificed after 8 weeks. There were no significant body weight differences between the animals with and without s.c. tumors at the time of sacrifice.
